# Improving the predictive accuracy of efficacy evaluation using tumor orthotopic transplant and resection model

**DOI:** 10.3389/fphar.2024.1309876

**Published:** 2024-02-27

**Authors:** Xiaoxi Li, Lingli Luo, Hui Qian

**Affiliations:** Department of Laboratory Medicine, School of Medicine, Jiangsu University, Zhenjiang, Jiangsu, China

**Keywords:** preclinical efficacy evaluation, tumor drug sensitivity analysis, OS benefit, efficacy evaluation model, tumor local recurrence and distant metastasis, tumor orthotopic transplant and resection model, neoadjuvant therapy and adjuvant therapy

## Abstract

Preclinical efficacy evaluation and tumor drug sensitivity analysis are two main applications of efficacy evaluation. Preclinical efficacy evaluation is to predict whether candidate drugs or therapies may improve patient outcomes in clinical trials. Tumor drug sensitivity analysis is an approach for the personalized evaluation and optimization of approved anti-cancer drugs and treatment regimens. Overall survival (OS) is the gold standard to evaluate the outcome of drugs or therapies in both clinical trials and clinical treatment. Many efficacy evaluation models, such as cell model, tumor cell-line transplant model, patient-derived tumor xenograft model, tumor organoid model, have been developed to assess the inhibitory effect of tested drugs or therapies on tumor growth. In fact, many treatments may also lead to malignant progression of tumors, such as chemotherapy, which can lead to metastasis. Therefore, tumor growth inhibition does not necessarily predict OS benefit. Whether it can prevent or inhibit tumor recurrence and metastasis is the key to whether drugs and therapies can improve patient outcomes. In this perspective, we summarize the current understanding of the pathological progression of tumor recurrence and metastasis, point out the shortcomings of existing tumor transplant models for simulating the clinical scenario of malignant progression of tumors, and propose five improved indicators for comprehensive efficacy evaluation to predict OS benefit using tumor orthotopic transplant and resection model. Improvement in the accuracy of efficacy evaluation will accelerate the development process of anti-cancer drugs or therapies, optimize treatment regimens to improve OS benefit, and reduce drug development and cancer treatment costs.

## Introduction

The development of anti-cancer drugs or therapies is a long, costly, and high-risk process and nine out of ten candidate drugs or therapies fail in clinical trials (Dowden and Munro, 2019). Improving the accuracy of efficacy evaluation can help increase the success rate of candidate drugs or therapies that have entered clinical trials, thereby reducing the cost of clinical trials and drug development. In addition to candidate anti-cancer drugs, efficacy evaluation can also be used for the evaluation and optimization of approved anti-cancer drugs and treatment regimens. Due to the complexity of factors affecting drug efficacy, directly testing the efficacy of drugs on patient-derived tumor cells or tissues, known as tumor drug sensitivity analysis, is the most direct and reliable approach to identify sensitive drugs and optimize treatment regimens. With the development of sequencing technology and cancer genomics research, a large number of high-frequency variant genes have been identified. Cancer genomics study has deepened our understanding of the pathological mechanism of tumorigenesis, and also discovered a lot of potential drug targets, which provides a theoretical basis for cancer precision diagnosis and individualized treatment. However, there are many factors that affect therapeutic response and clinical outcomes, and the intrinsic factor, genetic variations carried by tumors, cannot accurately predict therapeutic response, especially chemotherapy drugs.

Chemotherapy, radiotherapy, and surgery are important treatment methods for malignant tumors. However, many clinical studies have found that treatment can cause metastasis of various tumors. On the one hand, a large number of preclinical and clinical observations have found that cancer treatment can lead to an increase in circulating tumor cells, potentially inducing distant metastasis. On the other hand, treatment can cause a series of systemic host responses, which promote malignant progression of tumors, such as metastatic colonization, by affecting fibroblasts, immune cells, vascular endothelial cells in the tumor microenvironment. A recent study found that chemotherapy promotes tumor metastasis by inducing immunosuppression in the pre-metastatic microenvironment (Monteran et al., 2022). It is important to note that there are significant differences between clinical studies and preclinical tumor models, including tumor progression and treatment. For example, in clinical, a combination of drugs that enhance immunity is often used, which partially mitigates and counteracts the side effects of chemotherapy and its ability to promote tumor metastasis. However, in preclinical tumor models, the effects of single anti-tumor drug are often tested. Therefore, the results of clinical studies and preclinical tumor model research need to be carefully discussed and validated. As the mechanism of treatment-induced metastasis is beyond the scope of this perspective, we will not discuss it in depth. Several recent reviews have discussed in detail the current clinical observations, potential molecular mechanisms, and prevention strategies for treatment-induced metastasis (Karagiannis et al., 2019; Shaked, 2019; Su et al., 2023).

Another example is the ketogenic diet. Ketogenic diet, also called keto diet for short, is a high-fat, low-carbohydrate, and moderate-protein diet that forces the body to use ketones as its primary energy source instead of glucose from carbohydrates. Ketogenic diet is not only believed to be helpful for weight control, but also found to have the effect of inhibiting tumor growth (Kanarek et al., 2020; Steck and Murphy, 2020; Taylor et al., 2022), which may be some beneficial effects after the metabolic balance is disrupted. However, the metabolic imbalance caused by the ketogenic diet can also produce side effects, such as promoting cachexia. A recent study shows that the ketogenic diet can inhibit tumor growth in animal models, but it also leads to a shortened survival time in mice (Ferrer et al., 2023).

These observations suggest that whether tumor growth is inhibited is not sufficient to predict whether treatment can improve prognosis and prolong survival time. Due to limitations in tumor models and efficacy evaluation, the impact of treatment on tumor recurrence and metastasis is often overlooked, and relevant experimental studies and recognized evaluation methods have not been widely established. The important reason for us to write this perspective article is to provide new insights and reflections on the evaluation of antitumor drug efficacy from the perspective of optimizing preclinical tumor models, and to provide a theoretical basis for research methods on modeling tumor recurrence and metastasis. Comprehensive indicators for efficacy evaluation in tumor animal models, such as those evaluating metastasis and cachexia, should be developed and widely applied in preclinical efficacy evaluation.

The malignant progression of tumors and its impact on patients are important factors that determine the overall survival (OS). Cancer is a progressive disease, and when tumors relapse and metastasize, their molecular characteristics are often different from those of the primary tumor. Meanwhile, tumor recurrence and metastasis have a significant impact on the patient’s body, such as immune suppression, cachexia, and so on, which weaken the patient’s anti-tumor immune function and lead to treatment failure. The current experimental models used for efficacy evaluation mainly focus on whether drugs can inhibit tumor growth, and do not evaluate whether the tested drugs or therapies can prevent or inhibit malignant progression of tumors. Therefore, these models are not sufficient for predicting whether the tested drugs or therapies can improve OS in clinical trials and clinical treatment. A full understanding of tumor recurrence and metastasis and their impact on therapeutic response and clinical outcomes will help develop efficacy evaluation models that are closer to clinical scenarios, thereby improving the accuracy of preclinical efficacy evaluation and tumor drug sensitivity analysis.

It should be noted that lymphoma, although a type of blood cancer, can also form solid tumors in all parts of the body. The classification of lymphomas is relatively complex, and the current lymphoma classifications (Alaggio et al., 2022; Campo et al., 2022) are mainly based on three aspects: the cell of origin (COO) classification based on transcriptome profiles, the LymphGen classification based on genomic genetic variations, and the lymphoma dissemination. Similar to the metastasis of solid tumors, aggressive lymphoma often exhibits extranodal dissemination. Extranodal disseminations directly reflect the degree of lymphoma progression and affect the designation of treatment plans, making it one of the key prognostic indicators for lymphomas. Although the pathological mechanisms of metastasis and extranodal dissemination are largely different, similar to metastasis, extranodal dissemination is also a key event leading to poor prognosis in lymphoma patients. Because lymphoma has unique pathological mechanism, which is beyond the scope of this perspective, we will not discuss them in depth here. In our recent article (Li et al., 2023), we have discussed the pathological mechanism on extranodal dissemination of lymphoma, which may be helpful for the design of lymphoma mouse models for preclinical efficacy evaluation.

## The pathological progression of tumor recurrence and metastasis

**FIGURE 1:**
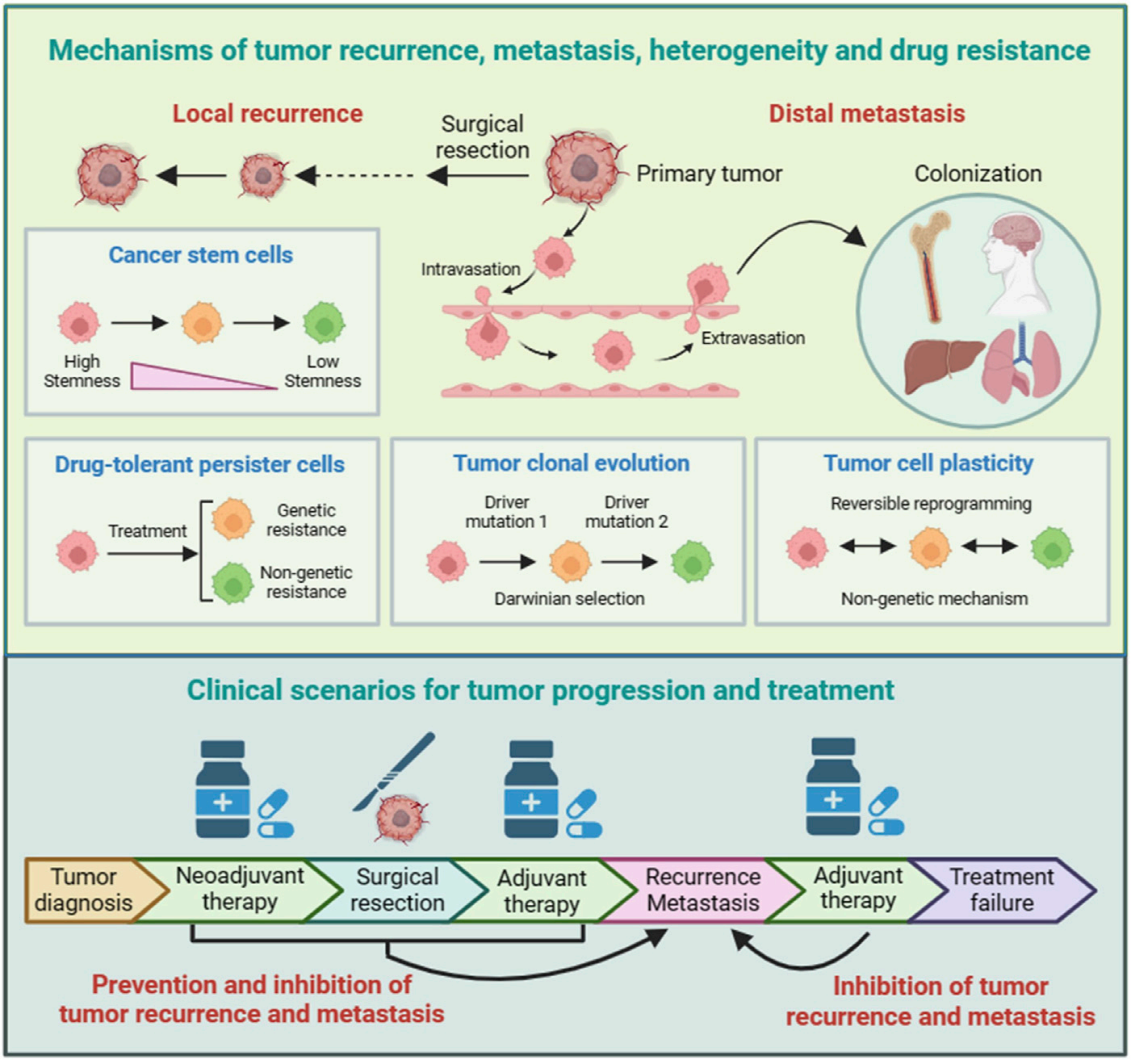
Mechanisms and clinical scenarios of tumor recurrence and metastasis. There are several models that help to understand the pathological mechanisms of tumor recurrence and metastasis. The cancer stem cells (CSCs) model and drug-tolerant persister cells (DTPs) model are two mechanisms of tumor recurrence and inherent drug resistance. The tumor clonal evolution model and tumor cell plasticity model serve as theoretical foundations for the selection and adaptation of tumor cells during tumor recurrence and metastasis, as well as for tumor heterogeneity and acquired drug resistance. For patients with early-stage tumors, surgical radical treatment can effectively prevent tumor spread by directly removing tumor tissues. For patients with late-stage tumors, some can first receive neoadjuvant therapy, and then undergo surgical treatment after the tumor has shrunk.

Tumor recurrence and metastasis are complex processes involving multiple steps (Klein, 2020; Gerstberger et al., 2023) (Figure 1). Metastasis mainly involves the following three steps. The first is the malignant transformation of tumor cells, mainly reflected in the acquired ability of tumor cells to invasion and metastasis, which enables tumor cells to leave the primary site and enter the lymphatic or blood circulation. The second is the adaptive selection of tumor cells in the lymphatic or blood circulation, where a small number of cells can eventually survive in the lymphatic or blood environment. These cells are also known as circulating tumor cells (CTCs). The third is the colonization of CTCs in distal tissues or organs. The colonization involves the adhesion of CTCs to lymphatic or vascular walls, extravasation, migration to a new location, and ultimately entering a state of rapid proliferation. Although CTCs can be easily detected in the blood of cancer patients, the process of how CTCs colonize in specific tissues and organs is still unclear. Many patients often develop multiple distant metastases within an uncertain period of time after surgical eradication and drug treatment. It is currently believed that when CTCs undergo colonization in a tissue or organ, the tumor cells enter a dormant state to avoid drug treatment or immune cell surveillance. When the time is right, such as after drug withdrawal or when the patient’s immune system weakens, the colonized tumor cells will rapidly grow to form new tumors. Therefore, eliminating CTCs or slowing their progression can prevent and inhibit tumor recurrence and metastasis, which is the key to improving patient outcomes. For example, surgical removal of the local tumor or systemic treatment that shrinks both the local tumor and distal tumors, rather than inhibiting tumor growth itself, can indirectly slow the progression of CTCs and serve as effective means of preventing and inhibiting tumor recurrence and metastasis.

There are several models that help to understand the pathological mechanisms of tumor recurrence and metastasis, as well as tumor heterogeneity and drug resistance, including cancer stem cells (CSCs) (Lytle et al., 2018; Skanderup and DasGupta, 2021), drug-tolerant persister cells (DTPs) (Phan and Croucher, 2020; Pu et al., 2023), tumor clonal evolution (Black and McGranahan, 2021; Rogiers et al., 2022), and tumor cell plasticity (Boumahdi and de Sauvage, 2020; Pérez-González et al., 2023) (Figure 1). Moreover, these models provide important insights for developing treatment strategies to overcome tumor recurrence and metastasis.

The CSCs model is based on tumor cells having different differentiation statuses. Tumor cells with lower differentiation and stronger stemness have a slower proliferation rate and can therefore resist chemotherapy and other therapies that kill rapidly dividing cells. The CSCs model provides important insights for tumor recurrence and drug resistance, and therefore, targeting CSCs represents a promising strategy to overcome tumor recurrence and drug resistance. The DTPs model is based on tumor cells having different proliferation statuses. A subset of cells in tumor are in a quiescent state, G0 phase in the cell cycle. Similar to CSCs, DTPs can also resist chemotherapy and other therapies that kill rapidly dividing cells. Despite the lack of specific markers, DTPs have specific transcriptional profiles and are therefore considered a group of inherent drug-resistant cells. Therefore, the identification of DTPs contributes to the development of therapies targeting DTPs to overcome tumor recurrence and drug resistance. Taken together, the CSCs model and DTPs model can be considered as theoretical foundations for tumor recurrence and inherent drug resistance.

The tumor clonal evolution model is based on tumor cells having distinctive genetic variations. Identification of driver genes and key events that drive tumor progression not only deepens our understanding of the pathological processes of tumors and the formation of tumor heterogeneity, but also helps to discover new targets for personalized cancer treatment. The tumor cell plasticity model emphasizes the reversible epigenetic changes that occur during tumor progression. Epigenetic modulation belongs to non-genetic mechanisms, and increasing evidence indicates that non-genetic mechanisms play key roles in tumorigenesis and drug resistance. Targeting epigenetic modulators has become a new category of anti-cancer drug development. Taken together, the tumor clonal evolution model and tumor cell plasticity model, as irreversible genetic mechanism and reversible non-genetic mechanism, respectively, serve as theoretical foundations for the selection and adaptation of tumor cells during tumor recurrence and metastasis, as well as for tumor heterogeneity and acquired drug resistance.

## Clinical scenarios for tumor progression and treatment

Tumor recurrence and metastasis are the main reasons for the failure of cancer treatment (Figure 1). Tumor recurrence refers to the phenomenon of tumor reappearance or spread after completion of treatment. There are many reasons for tumor recurrence, including incomplete tumor resection, emerging drug-resistant mutations, and decreased immune system function. Tumor metastasis mainly includes lymphatic metastasis, hematogenous metastasis, and implantation. The purpose of cancer treatment is to kill or slow the growth of cancer cells, thereby improving patient quality of life and overall survival. The selection of an appropriate therapy or treatment regimen depends on the type and stage of cancer, molecular classification, and the patient’s overall health and preferences.

For patients with early-stage tumors, surgical radical treatment can effectively prevent tumor spread by directly removing tumor tissues. For patients with advanced cancer, treatment strategies need to be personalized based on malignant degree of tumor. Common treatment strategies include direct surgery, surgery after neoadjuvant therapy, and non-surgical treatment. After surgery, patients with advanced cancer usually require adjuvant therapy or systemic treatment to prevent recurrence and metastasis.

Neoadjuvant therapy, also known as preoperative therapy, is a comprehensive treatment performed before surgery, including radiotherapy, chemotherapy, immunotherapy, targeted therapy and combined therapy. For patients who cannot undergo surgery, neoadjuvant chemotherapy can shrink the tumor and meet the surgical criteria. The purpose of neoadjuvant therapy is mainly to improve the radical cure rate of surgery through tumor downgrading, or to provide an opportunity for late-stage patients to undergo surgical resection.

If neoadjuvant chemotherapy fails to shrink the tumor to the surgical standards, then systemic treatment is expected to be the only feasible method to prolong the survival time of patients. Systemic therapy refers to the treatment of tumors through systemic drug therapy, immunotherapy, gene therapy, and other methods. Systemic treatment can cover tumor cells in various parts of the body, thereby reducing the risk of recurrence and metastasis, and improving the survival quality and survival time of patients.

In summary, the cancer treatment plan not only takes into account the overall health and preferences of each individual, but also needs to be adjusted according to the progression of the tumor. Whether it can prevent and slow tumor recurrence and metastasis is the key effectiveness indicator of various cancer treatment therapies and the goal of cancer treatment.

## Overall survival and progression-free survival

Progression-Free Survival (PFS) and Overall Survival (OS) are two important indicators commonly used in clinical trials to evaluate the therapeutic effectiveness and prognoses of cancer patients. PFS refers to the time from when a patient starts receiving treatment until the disease progresses or recurs. Specifically, PFS is used to assess whether a cancer patient’s disease is under control after receiving a certain treatment plan and how long this control can last. In contrast, OS refers to the duration from the initiation of treatment to the patient’s death due to any cause.

OS is a comprehensive indicator that takes into account all factors that may lead to the patient’s death, including disease progression, recurrence, and side effects of treatment. Although there is a certain relationship between PFS and OS, they are not entirely equivalent. The prolongation of OS indicates an extension in the patient’s overall survival time, which is the ultimate goal in assessing treatment effectiveness and prognosis. A longer PFS usually indicates a longer OS and a good prognosis, but it is not absolute. Sometimes, even if PFS is prolonged, OS may not be significantly improved due to the influence of other factors.

Compared to OS, there are fewer factors that affect PFS, making it a more direct indicator to reflect the effectiveness of treatment. Therefore, many clinical studies (Korn and Crowley, 2013; Belin et al., 2020; Gyawali et al., 2022) have begun to adopt PFS as the key indicator for prognostic evaluation instead of OS. However, it is worth noting that the accuracy of PFS in evaluating treatment outcomes can be influenced by other factors, such as individual differences in tumor malignancy. After receiving the same treatment, patients with low tumor malignancy tend to benefit more from the treatment and achieve longer PFS compared to those with high tumor malignancy. Therefore, individual differences among tumor patients are an important factor that must be considered when interpreting PFS and assessing drug efficacy.

In summary, both OS and PFS are important prognostic indicators that need to be evaluated and considered in the design of preclinical tumor models and the assessment of treatment effectiveness. It is also highly worthwhile to explore the question of how drug intervention or tumor progression ultimately determines OS and PFS.

## Efficacy evaluation models assessing tumor growth and metastasis

**FIGURE 2:**
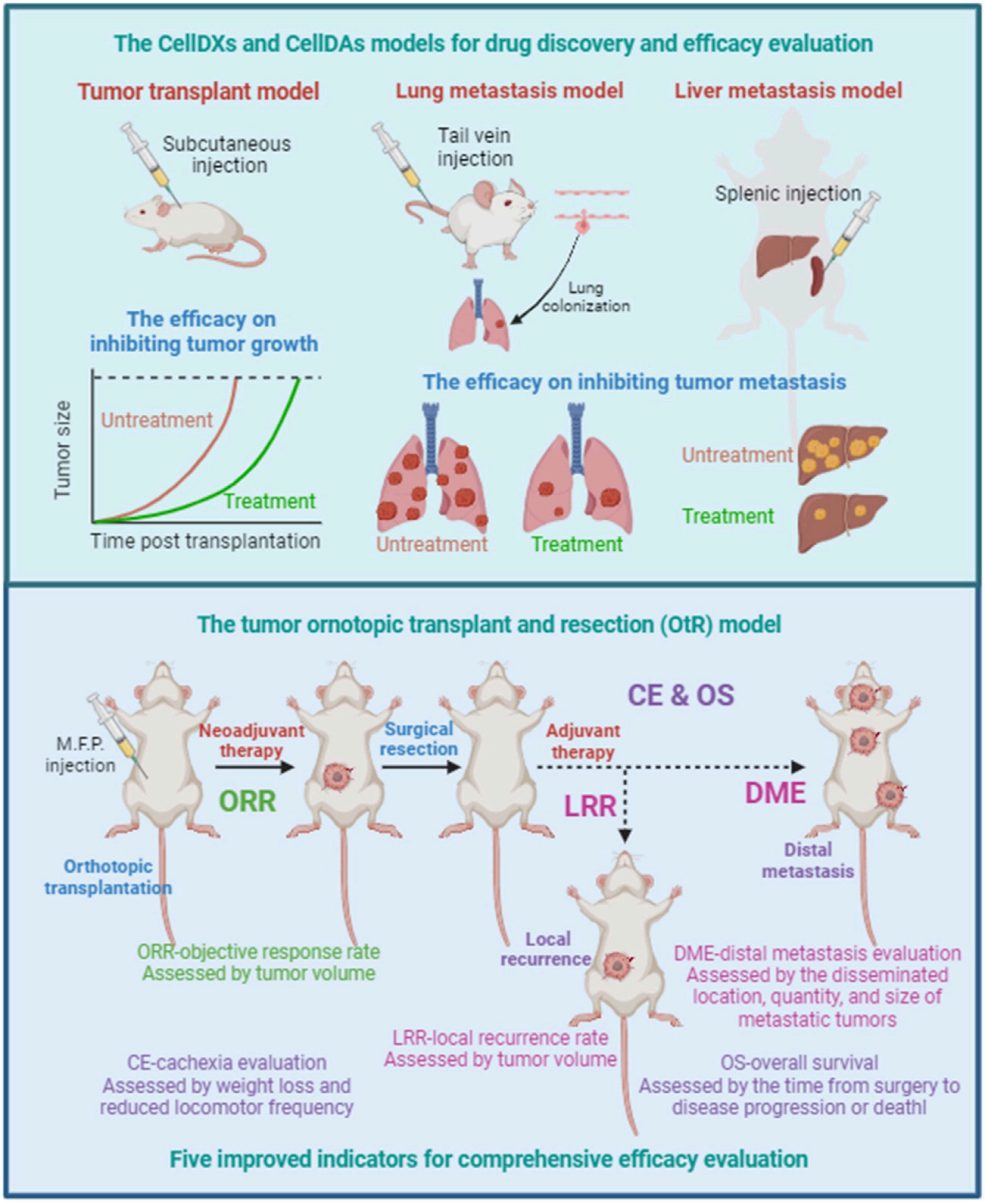
Efficacy evaluation for tumor growth, local recurrence, distal metastasis. Tumor cell-line transplant models are mainly designed to evaluate the inhibiting effectiveness of drugs or therapies on tumor growth, including human cell-line derived xenografts (CellDXs) model and murine cell-line derived allografts (CellDAs) model. Tumor metastasis models can be used to evaluate the effectiveness of drugs or therapies in preventing and inhibiting tumor metastasis, including the tail vein injection-mediated lung metastasis model and the spleen injection-mediated liver metastasis model. The tumor orthotopic transplant and resection (OtR) model, consisting of tumor cell-line orthotopic transplant models with surgical resection, can simulate clinical scenarios at different stages of tumor progression. Five key evaluation indicators for comprehensive efficacy evaluation are expected to improve the reliability of efficacy evaluation and the accuracy for predicting OS benefits. ORR, objective response rate. LRR, local recurrence rate. DME, distal metastasis evaluation. CE, cachexia evaluation. OS, overall survival.

Efficacy evaluation models for anti-cancer drugs or therapies mainly include cell model, tumor cell-line transplant model, patient-derived tumor xenograft (PDX) model, tumor organoid model. These efficacy evaluation models are mainly designed to evaluate the inhibiting effectiveness of drugs or therapies on tumor growth (Figure 2). The cell model cannot simulate the complex *in vivo* environment and is mainly used for preliminary validation of effectiveness. Tumor cell-line transplant model is the most commonly used efficacy evaluation model, including human cell-line derived xenografts (CellDXs) model and murine cell-line derived allografts (CellDAs) model (Wakefield et al., 2023). CellDAs model features a complete immune system and belongs to syngeneic transplant model, making it suitable for evaluating the effectiveness of immunotherapies such as antibodies and adoptive cell therapies. Although tumor cell-line transplant model can simulate the *in vivo* environment, the limited number of transplantable tumor cell lines and their relatively homogeneous genetic background cause tumor cell-line transplant model to be unable to simulate individual differences and intra-tumor heterogeneity.

PDX model is a transplant tumor model formed by implanting tumor tissues and primary cells from patients into immune-deficient mice, thus maximizing individual genetic differences. However, due to the lake of a complete immune environment, PDX models cannot be used for the evaluation of immunotherapy. Compared with PDX models, tumor organoid models have better repeatability, lower cost, stronger operability. However, similar to PDX models, tumor organoid models cannot be used for the evaluation of immunotherapy. In addition, autochthonous model can highly simulate tumor progression, including genetically engineered mouse models (GEMMs) and carcinogen-initiated mouse models. However, due to the high-cost, time-consuming and high individual differences, autochthonous model are not suitable for efficacy evaluation, mainly used for studying pathological mechanisms.

In some CellDAs models, changing the transplantation method can establish tumor metastasis models, which can be used to evaluate the effectiveness of drugs or therapies in preventing and inhibiting tumor metastasis (Figure 2). The injection of tumor cells directly into the bloodstream through the tail vein injection can simulate the processes of tumor cell circulation in the blood and tumor cell colonization. The lungs are the main colonization site in tail vein injection-mediated tumor metastasis model. The murine breast cancer cell line 4T1 and the murine melanoma cell line B16-F10 are the commonly used murine cell lines to establish lung metastasis models, which are used to simulate human breast cancer lung metastasis and melanoma lung metastasis, respectively. In addition, spleen injection is also a commonly used method to establish liver metastasis models that simulate the process of tumor cells metastasizing from the spleen to the liver.

However, lung metastasis model mediated by tail vein injection is very different from clinical scenarios in many aspects. Firstly, blood metastasis is not the main mode of metastasis of malignant tumors. Lymphatic metastasis, in fact, is the most common tumor metastasis for solid tumors. Secondly, the lung is not the main dissemination site of breast cancer. Brain, bone, liver, stomach, intestine, skin are the most common dissemination sites of malignant tumors. Finally, these metastases were not formed after drug treatment, and did not undergo selection and adaptation under long-term drug pressure. The spleen injection-mediated liver metastasis model also has similar shortcomings. Therefore, despite its strong tractability, tumor metastasis models are still insufficient to simulate clinical scenarios.

## Tumor orthotopic transplant and resection model

Considering the limitations of tumor cell-line transplant models and tumor metastasis models in simulating clinical scenarios, we propose a strategy for efficacy evaluation, which combines tumor cell-line orthotopic transplant models with surgical resection, referred to as tumor orthotopic transplant and resection (OtR) model (Figure 2). The OtR model can simulate clinical scenarios at different stages of tumor progression. Next, we will introduce the strategy of the OtR model using the 4T1 cell line as an example and explain its application in efficacy evaluation. The 4T1 cell line is a murine cell line model for triple-negative breast cancer (TNBC), originally derived from BALB/c strain mice. After being inoculated into the mammary fat pad of mice, 4T1 cells can grow rapidly in the mammary glands of the recipient mouse. Moreover, some tumor cells can metastasize to other sites through the lymphatic and blood systems, including the brain, bone, lungs, intestines, skin, and others. Therefore, the process of tumor progression in the 4T1 orthotopic transplant model is highly similar to the clinical scenario.

When the orthotopically transplanted tumor in the 4T1 model reaches a certain size, the primary tumor can be resected surgically to simulate clinical surgical resection (Figure 2). If the tumor is resected at a relatively small size, distant metastasis will occur in the recipient mice after a certain period of time, simulating the clinical scenario of tumor metastasis. If the tumor is resected at a relatively large size, not only distant metastasis but also local recurrence will occur in the recipient mice after a certain period of time, simulating clinical scenarios of both local recurrence and metastasis. Based on the OtR model, drugs and therapies can be administered before and after surgical resection to simulate clinical scenarios of neoadjuvant and adjuvant therapy and evaluate their effectiveness in preventing and inhibiting local recurrence and distant metastasis.

The OtR model is not only suitable for preclinical efficacy evaluation but also for evaluating and optimizing approved drugs or treatment regimens, such as the dosage, drug combinations, and drug administration sequences. Besides the 4T1 cell line, the EO771 cell line, originally derived from C57BL/6 strain mice, is also a murine cell line model to establish CellDAs model of breast cancer. Recently, the orthotopic transplantation model established by 4T1 and EO771 was used to study the mechanism of breast cancer metastasis (Yofe et al., 2023) and the effects of chemotherapy on the pre-metastatic microenvironment (Monteran et al., 2022). In addition, 4T1 cells can also be inoculated into specific lymph nodes to simulate the process of tumor metastasis to specific organs through the lymphatic system (Leslie et al., 2019), which can be further developed into the tumor lymph node transplant and resection (LNtR) model.

## Improving indicators for comprehensive efficacy evaluation

The main goal of cancer therapy is to prolong survival time and improve quality of life. In clinical trials, overall survival (OS) is the most reliable assessment criterion. Additionally, factors such as progression-free survival (PFS), objective response rate (ORR), and quality of life are also key indicators for evaluating the clinical efficacy of anti-cancer drugs or therapies. Accurate and reliable efficacy evaluation not only requires tumor animal models to be similar to clinical cancer treatment scenarios but also requires objective and reasonable evaluation indicators to comprehensively and accurately analyze experimental results. Here, we propose five key evaluation indicators: ORR-objective response rate, LRR-local recurrence rate, DME-distal metastasis evaluation, CE-cachexia evaluation, and OS-overall survival (Figure 1).

Objective response rate (ORR) refers to the efficacy of neoadjuvant therapy in inhibiting tumor growth before surgery, assessed by tumor volume. Local recurrence rate (LRR) refers to the preventive and inhibitory effects of neoadjuvant therapy and/or adjuvant therapy on local recurrence within a certain period of time, assessed by tumor volume. Distal metastasis evaluation (DME) refers to the preventive and inhibitory effects of neoadjuvant therapy and/or adjuvant therapy on tumor metastasis within a certain period of time, assessed by the disseminated location, quantity, and size of metastatic tumors. Cachexia evaluation (CE) refers to the overall side effect, assessed by weight loss and reduced locomotor frequency. Overall survival (OS) refers to the overall treatment efficacy, assessed by the time from surgery to disease progression or death. These five efficacy evaluation indicators can be assessed in the tumor OtR model, and the improvement of any one or several indicators suggests a certain degree of effectiveness.

In summary, the effectiveness of treatment is influenced by many factors, and efficacy evaluation is always very challenging. Efficacy evaluation models highly resembling clinical scenarios and objective, reasonable, and comprehensive efficacy evaluation indicators are the keys to improving the reliability of efficacy evaluation and the accuracy for predicting OS benefits.

## Discussion

Tumor is a highly heterogeneous disease, and the composition of tumor cells continuously evolves during tumor progression. The mechanism of antitumor drugs is also very complex, which can not only directly act on tumor cells but also indirectly affect tumor progression by acting on other cells in the tumor microenvironment. Tumor heterogeneity, molecular classification, tumor microenvironment, and tumor immunosurveillance are also key factors that affect tumor progression and therapeutic response.

Chemotherapy-induced metastasis and cachexia are the two most common observations that directly affect patient prognosis, however the effect of chemotherapy on metastasis and cachexia are fully underexplored. The five key evaluation indicators we proposed in this perspective will help to evaluate malignant characteristics such as metastasis and cachexia, thereby facilitating to identify the effect of chemotherapy on metastasis and cachexia. However, unlike tumor growth, modeling and evaluation of tumor metastasis and cachexia are not easy. In the OrR model, the incidence of metastasis is largely affected by individual differences and surgical procedures. For cachexia, weight loss is only one of the most obvious manifestations of cachexia and is the easiest to measure, but it is not a specific indicator of cachexia. For example, gastrointestinal tumors can also lead to weight loss due to difficulties in eating and digestion. Therefore, establishing more uniform modeling methods and more accurate evaluation indicators is an important direction for the OtR model in the future. With the development and improvement of the OtR model, we believe that identifying the effects of drugs on metastasis/cachexia and developing intervention strategies to prevent metastasis/cachexia, will gradually become the most important topic for the development of anti-cancer drugs and therapies in the future.

In recent years, immunotherapy has shown promise in clinical trials and clinical treatment, suggesting patient’s immunity should be a key indicator predicting patient outcomes. Therefore, evaluation indicators of patient’s immunity should be developed in efficacy evaluation. It is worth noting that the CellDAs model belongs to syngeneic transplant model, and can therefore also be used for evaluating the efficacy of immunotherapy. Due to the numerous types of anti-cancer immunotherapies, each type of immunotherapy has its own characteristics. Therefore, the design and evaluation indicators of preclinical animal models for evaluating the efficacy of immunotherapy are very complex and challenging, which is beyond the scope of this perspective.

From this perspective, we take the 4T1 orthotopic transplant model as an example to represent the combination of the orthotopic transplant model and surgical resection to resemble clinical scenarios of tumor recurrence and metastasis. However, there are very limited murine tumor cell line models that can be used for orthotopic transplantation and exhibit high-frequency metastasis. In the future, murine tumor cell lines with genetic variants similar to those found in human tumors should be developed to enhance the diversity of efficacy evaluation models. Genetically engineered mouse models (GEMMs) and carcinogen-initiated mouse models are precious resources for the discovery and establishment of new murine tumor cell lines.

In summary, in this article, we have discussed the rationality of currently used preclinical tumor models for evaluating drug efficacy, proposing that preventing and inhibiting tumor recurrence and progression, rather than tumor growth, is the key objective for improving the prognosis of cancer patients with antitumor therapies. Furthermore, we discussed the limitations of common preclinical tumor models in efficacy evaluation and proposed the tumor orthotopic transplant and resection (OtR) model as a new strategy to simulate tumor recurrence and metastasis. Finally, we suggested five indicators for comprehensive efficacy evaluation to assess the effectiveness of antitumor therapies in inhibiting tumor progression. The strategies and indicators for efficacy evaluation proposed in this perspective will provide new insights for the optimization of preclinical tumor models and improving the predictive accuracy of efficacy evaluation.

## Data Availability

The original contributions presented in the study are included in the article/Supplementary material, further inquiries can be directed to the corresponding authors.
